# Nitroglycerin as a Treatment Modality for Recurrent Isolated Paracentral Acute Middle Maculopathy: A Case Report

**DOI:** 10.7759/cureus.20447

**Published:** 2021-12-15

**Authors:** Jason K Sendek, Anita Goyal, Robert G. Josephberg

**Affiliations:** 1 Ophthalmology, Briarcliff High School, Briarcliff Manor, USA; 2 Ophthalmology, New York Medical College, Valhalla, USA

**Keywords:** nitroglycerin, retina, lesion, scotoma, pamm

## Abstract

Paracentral acute middle maculopathy (PAMM) is a condition characterized by ischemia affecting the deep capillary plexus layer of the macula, often resulting in a paracentral visual scotoma. PAMM has been reported in association with retinal vascular diseases or as an isolated phenomenon in young and healthy individuals. There is currently no treatment for PAMM. We report a case in which sublingual nitroglycerin reversed developing visual scotomas, or blind spots, in a patient with known recurrent episodes of PAMM.

This case describes a male patient with previously documented evidence of PAMM in both eyes following episodes of extreme physical activity or dehydration. These episodes would often last days without ultimate resolution. After one such occurrence in his right eye, the patient was treated with a sublingual nitroglycerin tablet immediately after the development of new visual scotomas.

The patient’s visual symptoms improved within minutes of administering sublingual nitroglycerin, and completely resolved within hours.

## Introduction

Paracentral acute middle maculopathy (PAMM) is a condition characterized by acute onset of paracentral visual scotoma and is thought to be caused by ischemia of the middle retinal layers [1]. While there is no incidence rate in the United States, one study reported a 3.8% incidence rate (15/395) of patients who fit eligible criteria [2]. PAMM was first characterized in 2013, as a distinct hyperreflective band-like spectral-domain optical coherence tomography (OCT) lesion at the level of the inner nuclear layer. The lesion is typically seen with near-infrared reflectance imaging in the parafoveal region. PAMM lesions are thought to be caused by localized retinal capillary ischemia and can be the cause of persistent scotomas. These lesions often result in thinning and atrophy of the affected inner nuclear layer, accounting for the permanent visual defects that patients with PAMM typically experience [1]. PAMM may occur in conjunction with other retinal vascular disorders such as branch retinal vein occlusion, central retinal artery occlusion, or occur as an isolated entity in healthy, young individuals [3]. 

PAMM lesions occur in the parafoveal region of the macula, which is a region particularly susceptible to ischemic insults [4]. The oxygen demand of the macula is higher than any other region of the retina. Oxygen diffusion from the choroid to the retina is limited by retinal thickness, which is greatest parafoveally. The middle retinal layers of the parafoveal macula have been described as a watershed-like region, which has a high perfusion demand whose supply is limited by its structure, placing it at the greatest risk for an ischemic insult [5]. 

When PAMM is discovered in an apparently healthy individual with no risk factors, the individual should undergo a systemic work-up to rule out any underlying vascular disease [6]. Medications, such as amphetamines, caffeine, vasopressors, and oral contraceptives, have been reported in association with PAMM. In addition, migraines, severe hypovolemia, orbital compression injury, and viral illness have been linked to PAMM [7]. The clinical diagnosis of PAMM is based on a patient’s history of an acute onset of a negative scotoma with no other ocular symptoms. PAMM can develop in younger individuals with no ocular history, or without preceding visual symptoms. PAMM lesions may appear as subtle grey parafoveal lesions within the retina on fundoscopy. Funduscopic examination may not reveal the reason for the subtle scotomas as described by the patient; however, spectral-domain OCT imaging allows for the capturing of the findings on the detailed images of the retina [8].

Currently, there is no treatment for PAMM, but treatment for the underlying cause is often necessary. Symptomatic scotomas may fade but often are persistent [9]. With no definitive cure, new treatments must be tested to assess the efficacy to reduce the effects of ischemia. There are several reports that discuss the use of sublingual nitroglycerin as an effective therapy for episodes of acute loss of vision due to vascular occlusion. We hypothesized that in our patient, nitroglycerin could potentially play a role in reversal of retinal capillary ischemia and visual symptoms. 

Nitrates work on the muscle walls of the veins and arteries. When nitroglycerin enters the bloodstream, it is converted to nitric oxide, which acts on the veins and arteries. Nitric oxide is a powerful vasodilator that causes relaxation of the vascular tone [10]. This dilation affects blood flow, perfusion pressure, and flow resistance as these three systems are dependent on others. As the radius of the retinal blood vessels decrease or constrict, the perfusion pressure will decrease, slowing the flow of blood through the retina. When the radius of the retinal blood vessels dilate or increase, the opposite occurs.

In an article published in 1990 by Dr. Sharon Kuritzky, two patients are described with sudden total loss of vision from one of their eyes, treated effectively with 0.4 mg of nitroglycerin sublingually [11]. In 1983, a study was conducted by Wizemann et al., showing that nitroglycerin produced complete recanalization of experimentally produced retinal artery occlusion in rats [12]. In another study published in 1998, scientists induced branch vein occlusion in miniature pigs, and found that intravenous administration of the nitric oxide donor, sodium nitroprusside, reversed arteriolar vasoconstriction [13]. 

Nitroglycerin is usually a safe, widely used medication whose side effects are rapidly reversible. Previous studies have found that nitroglycerin may be efficacious in the treatment of acute retinal vascular conditions. This case report is unique in that it documents the first reported case of a proposed treatment for PAMM in a patient with recurrent episodes in both eyes of PAMM.

## Case presentation

**Figure 1:**
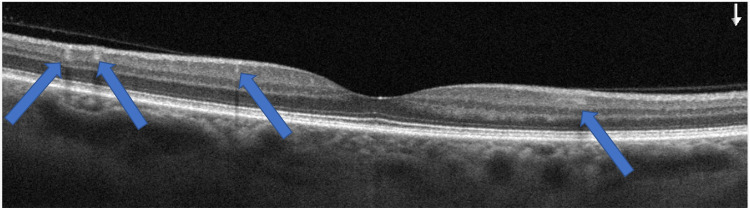
Image of the OCT B scan cross section shows the hyperreflective lesions (white bright spots) in the middle retina of the right eye OCT, optical coherence tomography

**Figure 2:**
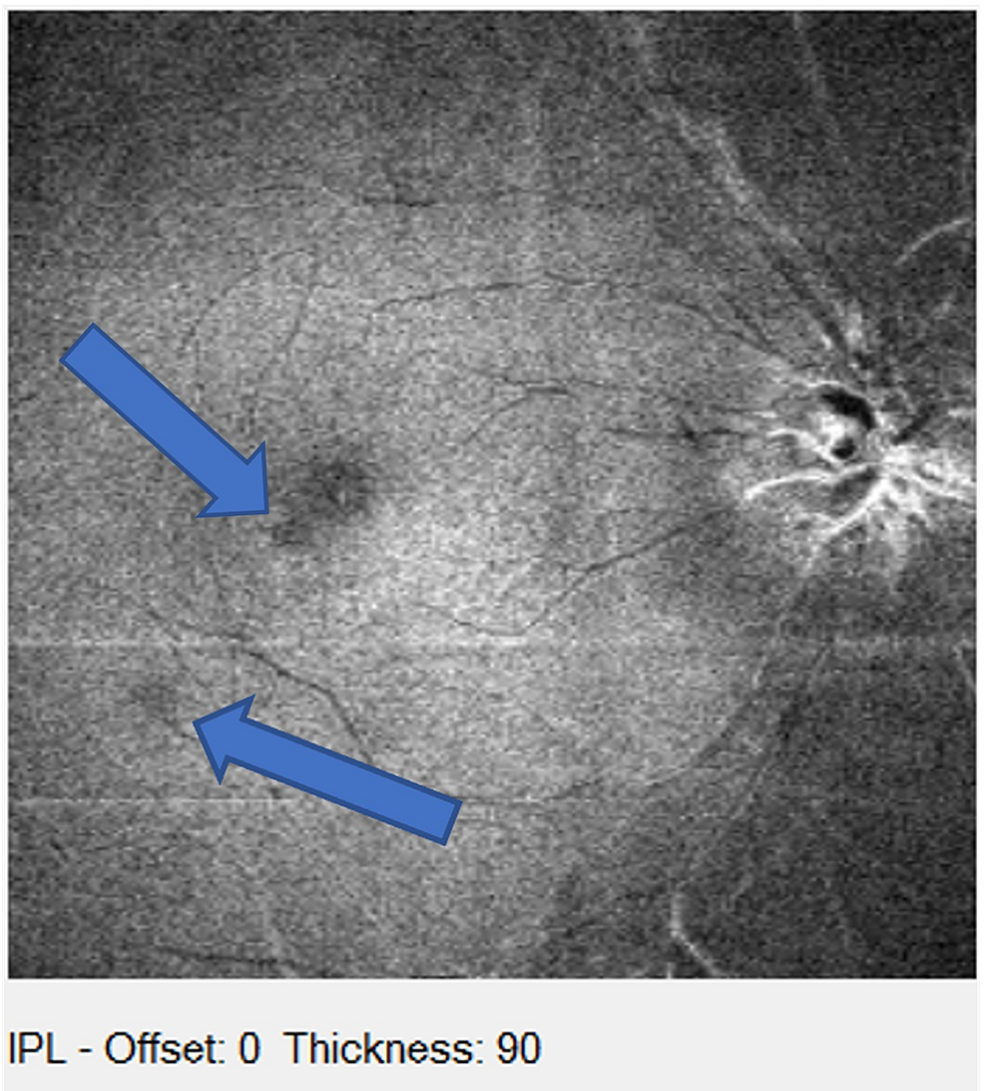
Images taken by the OCT en face technique shows the ischemic spots of the hyporeflective lesions (black spots) taken of the inner plexiform layer in the right eye OCT, optical coherence tomography

A 47-year-old Caucasian male with a history of an aortic valve replacement, Factor V Leiden anomaly, migraines, and a competitive cycling hobby presented with new paracentral blind spots in the right eye following a fishing trip in Florida on August 28, 2014. The patient reported that the vision loss began during a fishing trip when he became dehydrated and had not resolved. He described three to four similar events that occurred previously following episodes of extreme physical activity, however, all resolving. On presentation in 2016, visual acuity was 20/20 in both eyes. No fundus abnormalities were noted. Amsler grid testing revealed two scotomas about 1 and 4 degrees superior nasal to fixation in the right eye. Spectral-domain OCT imaging also revealed several hyperreflective bands in the middle retina of the right eye (Figure 1). In Figure 2, the hyporeflective lesions seen at the border of the fovea inferior temporal and slightly further out were consistent with his subjective superior nasal scotomas on Amsler grid testing. Spectral-domain OCT findings of PAMM were corroborated with the Chief of the Retinal Service at the New York Eye and Ear Infirmary. The patient was diagnosed with findings consistent with PAMM. At that time, no treatment was given. While diagnostic measures were not taken during this patient’s first few described episodes, it was thought that the previous episodes were also consistent with PAMM, given their similar presentation on the Amsler grid testing and symptomatology.

In mid-2018, the patient presented with a left-sided visual scotoma that had developed following a recent episode of febrile gastroenteritis. Treatment started with niacinamide OTC minerals, which helped initially over 30-40 minutes. After consulting with his cardiologist, nitroglycerin was prescribed but not taken. The patient was treated with 325 mg of aspirin and 200 mg of ibuprofen every 4 hours, with minimal relief. Aspirin and ibuprofen were administered because they are anti-inflammatory and blood thinners. The scotoma improved over the course of the next week but did not fully resolve. A permanent small scotoma remained.

In 2019, the patient began to experience similar symptoms after vigorous exercise. The patient was treated with 0.4 mg nitroglycerin sublingually previously prescribed by his cardiologist. Within 45 minutes, the visual scotoma improved, and within a few hours, it completely resolved. Since this occasion, the patient experienced two subsequent episodes of the development of visual scotomas, both resolving within minutes of the administration of sublingual nitroglycerin. 

The patient described the lesions as permanent obstructions in the field of vision. They appear as small opaque structures, with a purple hue. The structures blend into the background without a distinct outline. The right eye lesions seen in Figure 1 appeared at about 11 o’clock in the field of the patient's vision and did not move. 

## Discussion

PAMM was first characterized in 2013 as a lesion involving the middle layer (inner nuclear layer) of the retina. It was found to be a distinct entity from acute macular neuroretinopathy (AMN), although both were thought to be due to retinal capillary ischemia. With the advent of more sophisticated imaging systems, the finding of PAMM became more commonly encountered than AMN. In fact, PAMM has now been linked to numerous retinal vascular diseases, as well as an isolated, idiopathic finding that may even develop in young and healthy individuals. Once PAMM is discovered, a comprehensive search for contributing systemic or extrinsic vascular risk factors should be conducted. 

Previous studies have successfully increased blood flow in the retinal area. A 1993 study proved that both nifedipine and glyceryl trinitrate transiently increased blood flow velocity in the central retinal artery although neither drug candidate affected blood pressure or retinal resistive index [14]. Prostaglandin E1 was proven successful in treating non-arteritic posterior ischemic optic neuropathy, a disorder involving reduced blood flow [15]. A recent 2021 study of a 12-year-old male patient showed that nicotinic acid may improve retinal ischemia, an integral part of the treatment for a sight-threatening condition [10].

Our patient presented to his retina specialist with a complaint of persistent blind spots in and around his central vision that bothered the patient. As studies were undertaken to establish the diagnosis, spectral-domain OCT imaging revealed classic PAMM lesions; hyperreflective band-like abnormalities at the level of the inner nuclear layer, which corresponded to the patient’s description of his blind spots and congruent with Amsler grid testing with paracentral scotomas. While the diagnosis was felt to be clear, the etiology was still unknown. The patient subsequently underwent a comprehensive systemic work-up, including carotid artery imaging, echocardiogram, laboratory testing including lipid panel, A1C, erythrocyte sedimentation rate, C-reactive protein, and hypercoagulability testing. Aside from the known Factor V Leiden mutation, all testing were found to be within normal limits. Although the patient had a known aortic valve replacement, echocardiogram testing did not show mobile thrombi and was otherwise within normal limits. 

A meticulous clinical exam was conducted to rule out an occult retinal artery occlusion or retinal ischemia caused by a masquerading cilioretinal artery occlusion. Fluorescein angiography testing was conducted, showing possible deep retinal capillary ischemia in the corresponding area of the patient’s described scotomas, with no other retinal vascular anomalies noted. OCT angiography imaging was not performed. 

On detailed history and review of systems, the patient reported these recurrent episodes only after prolonged episodes of extreme activity or dehydration. He denied migraines as being related to these visual symptoms. Migraines were thought of and always as in the differential. However, in this case there was no history of headaches. While the aura of migraine can be present without headaches, the aura would be in two eyes together simultaneously, which is very common. Our patient did not have this. Also, a one-eye or monocular retinal migraine can simulate this, but rarely occurs so often and rarely leaves defects from the retinal spasm causing it. Furthermore, there were never signs of any retinal spasm associated with an isolated monocular migraine or retinal spasm when he was clinically seen during these episodes. The patient had a full neurological work-up, which discounted migraines as a cause. Certainly, nitroglycerin would make a classic migraine worse since it dilates vessels which are the initial cause of the headaches in migraine. The patient denies use of amphetamines and vasopressors. Consumption of caffeine was not found to be significant in relation to his visual symptoms; patient reported that he did not drink coffee or consume caffeine-containing products ever. It was thus concluded that the patient’s episodes of visual disturbance were likely due to a combination of factors, such as hypovolemia, physical exertion, and febrile illness. It is possible that the patient’s history of having Factor V Leiden mutation additionally contributed to his susceptibility to retinal capillary ischemia. 

One may hypothesize that amaurosis fugax was the cause of this patient’s previous episodes of visual scotoma; however, this hypothesis was rejected, as this patient had no history or findings of carotid artery disease, and several of his episodes resulted in permanent visual scotomas, unlike amaurosis fugax. 

Nitroglycerin is a potent vasodilator that has been studied in relation to retinal blood flow. Nitroglycerin administration in human subjects altered retinal vessel caliber significantly; sublingual administration of 0.64 mg nitroglycerin produced a highly significant dilatation in both retinal arteries (6%) and veins (5%). The authors report that retinal circulation supplying the parafovea is autoregulated in relation to perfusion pressure, and that a 5% change in retinal blood vessel caliber represents a 20% change in blood flow [16]. 

Studies have shown that nitrates, or nitric oxide donors, may cause a reversal of retinal vessel vasoconstriction. In a study performed on the effect of nitroprusside on arteriolar constriction after retinal branch retinal vein occlusion, it was found that preretinal intravitreous administration of the nitric oxide donor sodium nitroprusside reversed arteriolar vasoconstriction that occurred in parallel with the branch vein occlusion. The study suggests that nitric oxide donors may provide an efficient means for continuous delivery of nitric oxide to the retinal circulation, and therefore may significantly contribute to the restoration of arteriolar blood supply [13]. In our patient, it is hypothesized that nitroglycerin administration, through the effects of nitric oxide, reversed arteriolar and capillary plexus ischemia causing the characteristic inner nuclear layer lesions seen in PAMM. 

Another study sets out to answer if isosorbide mononitrate nitrate influences blood flow in the optic nerve head and choroid. The 12-subject study, following nitrate intake, showed optic nerve flow increase from baseline of 19.8% [17]. The profound effects caused by nitroglycerin appears to be the result of it working more on the veins rather than their arterial counterparts [18]. This is especially interesting because it was proven that the deep capillary plexus serves as a primary site of venous outflow, which happens to be a common location for PAMM lesions to develop. 

In our patient, the etiology of his recurrent paracentral visual scotomas was felt to be most consistent with PAMM. Although the patient does have a history of possible hypercoagulable state secondary to Factor V Leiden anomaly, a thromboembolic event was felt to be unlikely given normal fundus examination and fluorescein angiography. Presumably, carotid artery disease did not contribute as our patient had no preceding risk factors or history of such disease. Ocular migraine was additionally less likely given the permanent nature of one of his episodes of visual scotoma. Given that the patient had spectral-domain OCT imaging evidence of hyperreflective bands at the level of the inner nuclear layer, it is most likely that the patient’s recurrent episodes of visual scotomas are secondary to PAMM, thought to be precipitated by hypovolemia secondary to severe dehydration and/or severe physical exertion. At this point in the patient’s life, he has no other active medical problems. The prescription of nitroglycerin was consented to by the patient, and he was given the nitroglycerin prescription by his cardiologist. Nitroglycerin is approved by the FDA in the United States for cardiac conditions, and once approved can be prescribed off-label legally for any other conditions as with all other drugs similarly approved by the FDA. Also, there were no ethical concerns since everybody (patient and doctor) mutually agreed in writing and verbally on the decision.

## Conclusions

We report a unique case report of a patient with recurrent PAMM treated with sublingual nitroglycerin after the onset of visual scotomas. This report suggests that nitroglycerin may play a role in the reversal of retinal capillary ischemia and could potentially be used in the treatment of other ischemic vascular conditions. While this conclusion is not definitive solely from this report, we have shown that this sort of treatment has been effective on numerous other occasions as well, further supporting our conclusion. This case report’s purpose is to make other physicians aware of a possible treatment benefit. Underlying conditions matter, but none were treatable in this report. The patient’s cardiovascular history with an aortic valve replacement may have influenced the result outputs, but unlikely given the data. Hydration did not work, and most episodes were brought upon from demanding exercise, rather than dehydration. Further studies are needed to better establish the role of nitroglycerin in ischemic vascular conditions.
